# Supplementary material for: A myosin II nanomachine mimicking the striated muscle
Source: Nat Commun. 2018 Aug 30;9:3532. doi: 10.1038/s41467-018-06073-9 (PMC6117265; doi:10.1038/s41467-018-06073-9)
Supplement: Supplementary file 1 — Supplementary Information [file 41467_2018_6073_MOESM1_ESM.pdf]

Supplementary Information

A myosin II nanomachine mimicking the striated muscle

Pertici et al.

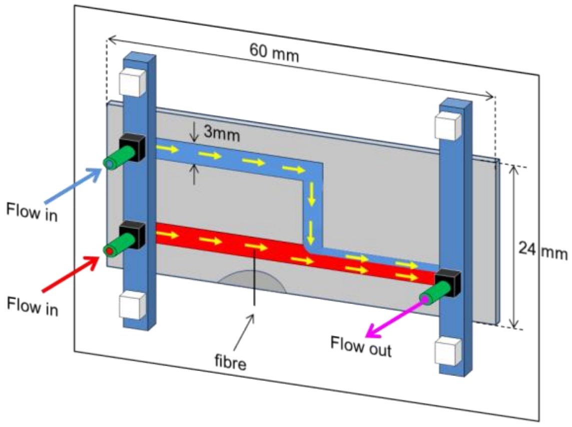

Supplementary Fig. 1. Schematic representation of the experimental chamber. The chamber is made by two coverglass slides separated by two layers of parafilm cut in order to obtain two separate 180-µm deep channels that converge in their final tract. The flow from the upper channel (blue) and that from the lower channel (red) remain separate at the convergence because fed with the same pressure. Yellow arrows indicate the direction of the flow. The chamber is sealed to withstand the fluid pressure by heating the two parafilm layers. The support for the HMM motors consists of a single mode optical fibre chemically etched to a diameter of ~ 4 µm and coated with nitrocellulose for HMM attachment. The support is placed in the lower channel near the convergence. HMM and ATP are flowed through the lower channel, BTA through the upper channel. A BTA, captured by the laser trap in the upper compartment, is brought in the proximity of the myosin array on the fibre by moving the micropositioner carrying the chamber–nanopositioner system. The dimensions of the flow chamber are reported in the figure.

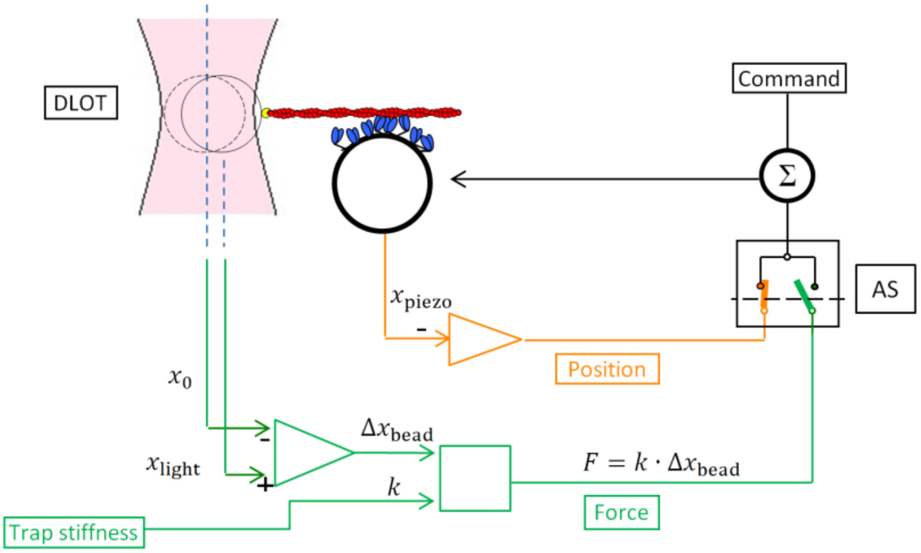

Supplementary Fig. 2. Block diagram of the system for recording/control the nanomachine mechanics. The analogue switch (AS) selects the signal that feeds the summing amplifier (Σ) to be compared with the command (black): either the position of the nanopositioner (x_{piezo}) carrying the support for the myosin array (blue) (position feedback, orange) or the force (force feedback, green). Force is calculated as the product of the stiffness of the trap (k) times the change in the position of the bead (Δx_{bead}) attached to the actin filament (red) via gelsolin (yellow), calculated from the change in light momentum (x_{light}) of the dual laser (pink), relative to that at zero force (x_0).

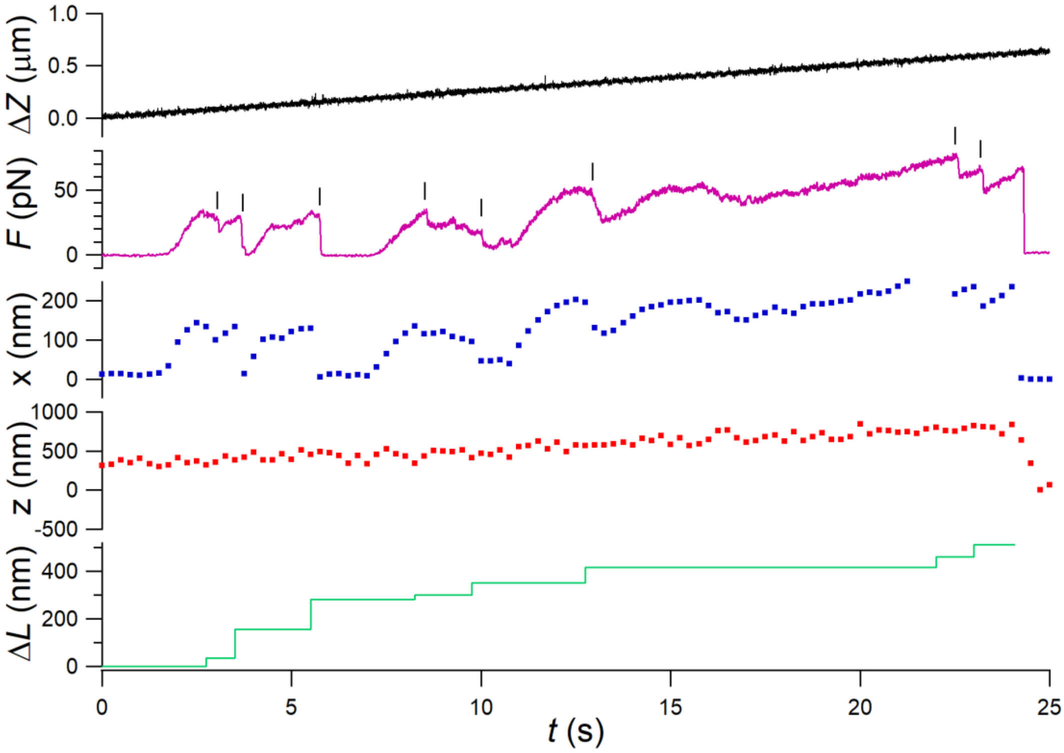

Supplementary Fig. 3. Extent of the array of interacting HMM estimated from rupture events. Movement of the nanopositioner away from the motor surface in the orthogonal direction (ΔZ, black) to stretch the rigor bonds. The x component of force (F, magenta) shows rises of F followed by abrupt drops (indicated by the small vertical bars) in correspondence of the rupture of rigor bonds. The bead movement along x- (blue) and z-axes (red) is measured using LabVIEW custom-made image analysis program kindly provided by Marco Capitanio. The compliance of the trap along the z-axis ($\geq$ 100 nm·pN^{-1}) is 30 times larger than that along the x-axis (3.7 nm·pN^{-1}), thus, along z-axis, rupture events are not detectable apart from the last one corresponding to the complete detachment of the filament from the motor array. The x component of the abrupt displacement towards the trap centre following the rupture of a rigor bond measures the distance travelled following rupture events (ΔL, green)[1]. In this experiment the sum of ΔL gives a total extent of the array of interacting motors of 510 nm. Acquisition rate: 500 points·s^{-1} (ΔZ and F) and 4 frames·s^{-1} (x and z). Motor array obtained with [HMM] = 100 µg·ml^{-1}.

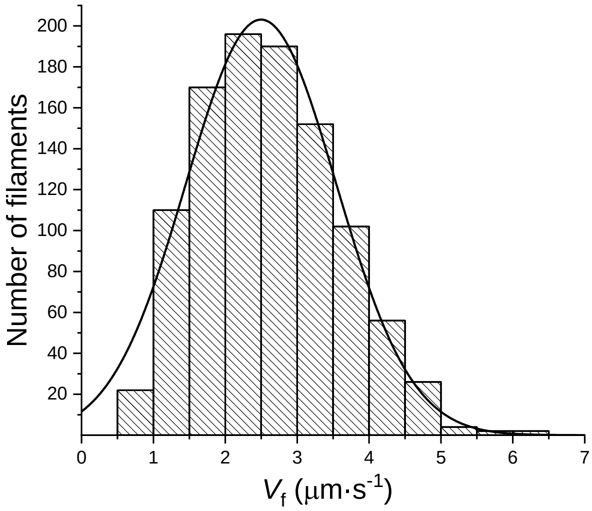

Supplementary Fig. 4. Frequency distribution of the sliding velocity of actin filaments (V_f) on HMM in IVMA. HMM were from the same preparations used for the machine mechanics. Temperature 23°C. V_f was grouped in classes of 0.5 µm·s^{-1}. The continuous line is the gaussian fit with centre 2.50 ± 0.05 µm·s^{-1} and σ 1.04 ± 0.05 µm·s^{-1}. Data are mean ± SEM.

a

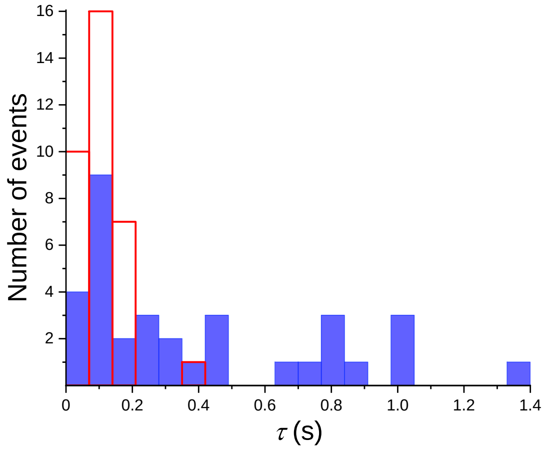

b

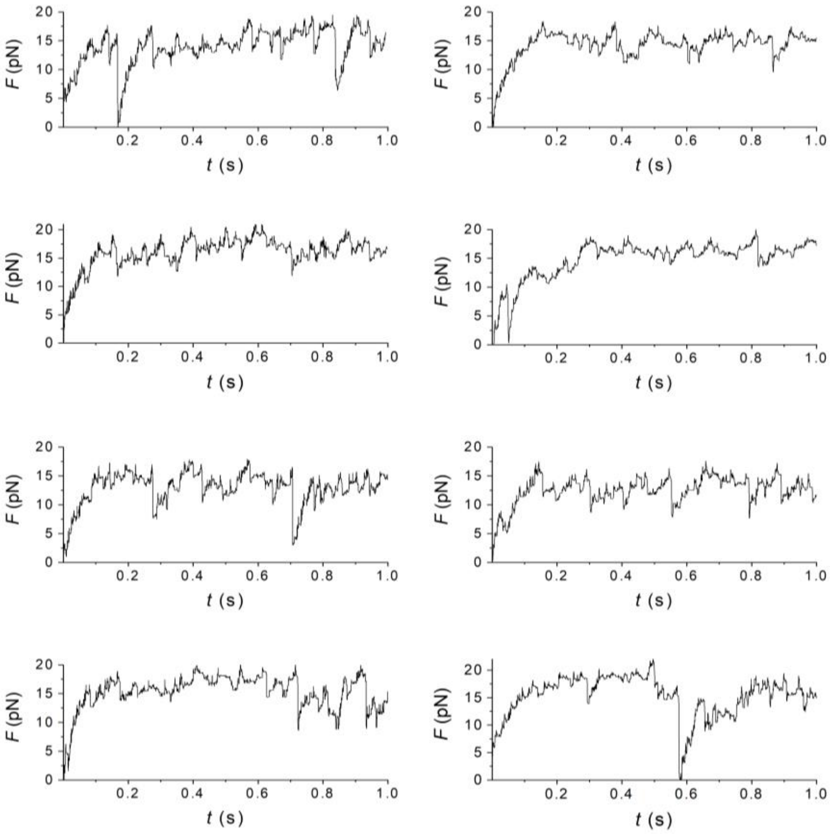

	Number of records	Number of steps to get to 0.95 F_0	Number of records with force drop to 0
viscosity coefficient = 1 mPa·s (no methylcellulose)	72	75.5 ± 2.7	14
viscosity coefficient = 7 mPa·s (0.5% methylcellulose 400 cP)	68	72.5 ± 5.1	20

Supplementary Fig. 5. Simulation of force development and of the effect of medium viscosity. **a** Frequency distribution of the τ of force development in position clamp. τ is estimated from the fits to the recorded force traces as in Fig. 3a,b (blue, n = 34) and to the simulated force traces as in

Fig. 3c,d (red, n = 34), without distinction between initial force rise and force redevelopment. τ for the recorded traces is calculated excluding the values > 0.4 s (which are clearly affected by pauses and fluctuations in the force) and is 0.15 ± 0.09 s. τ for the simulated traces is 0.11 ± 0.06 s. Data are mean ± SEM. **b** Simulated force development against the trap compliance by an array of motors with N = 16 in a medium with viscosity coefficient 1 mPa·s (control, left column) and 7 mPa·s (to simulate 0.5% w/v methylcellulose solution, right column). Traces occasionally show a transient drop of force to 0, marking loss of acto-myosin interaction. The table reports the statistics about the number of interactions occurring during the time (= 3τ) necessary to get 0.95 F_0 and the number of records with force drop to 0 either in the control simulation (upper row) and in increased viscosity coefficient run (lower row). In the absence of force drop to 0 (3/4 of records), on average 75 interactions or steps are necessary to get 0.95 F_0, in agreement with the number of consecutive steps allowed in probabilistic terms with N = 16. In fact, the probability P of x consecutive steps depends on N according to the equation $P(x, N) = [1-(1-R)$^$(N-1)]$^$(x-1)$, where R is the duty ratio. With τ = 0.11 s (legend of Fig. 3) and F_0 = 16 pN (Fig. 2e), the initial rate of force rise is 145 pN·s^{-1} and the initial V is 0.538 μm·s^{-1}. At this V, from the F-V relation (Fig. 2e) and the N_a-F/F_0 relation (Fig. 4e) it results a number of attached motors N_a of 3.7 and a R (= 3.7/16) of 0.24. With N = 16 and R = 0.24 the equation gives a number of consecutive steps x of 50. However, it must be considered that R = 0.24 is the lower limit of the values experienced during force development (as it is calculated on the initial highest rate of force rise). Taking the average R between the initial value and the F_0 value, we obtain ((0.24+0.32)/2=) 0.28 and x becomes 137, which is larger than the value of 75 obtained from the simulation of force development.

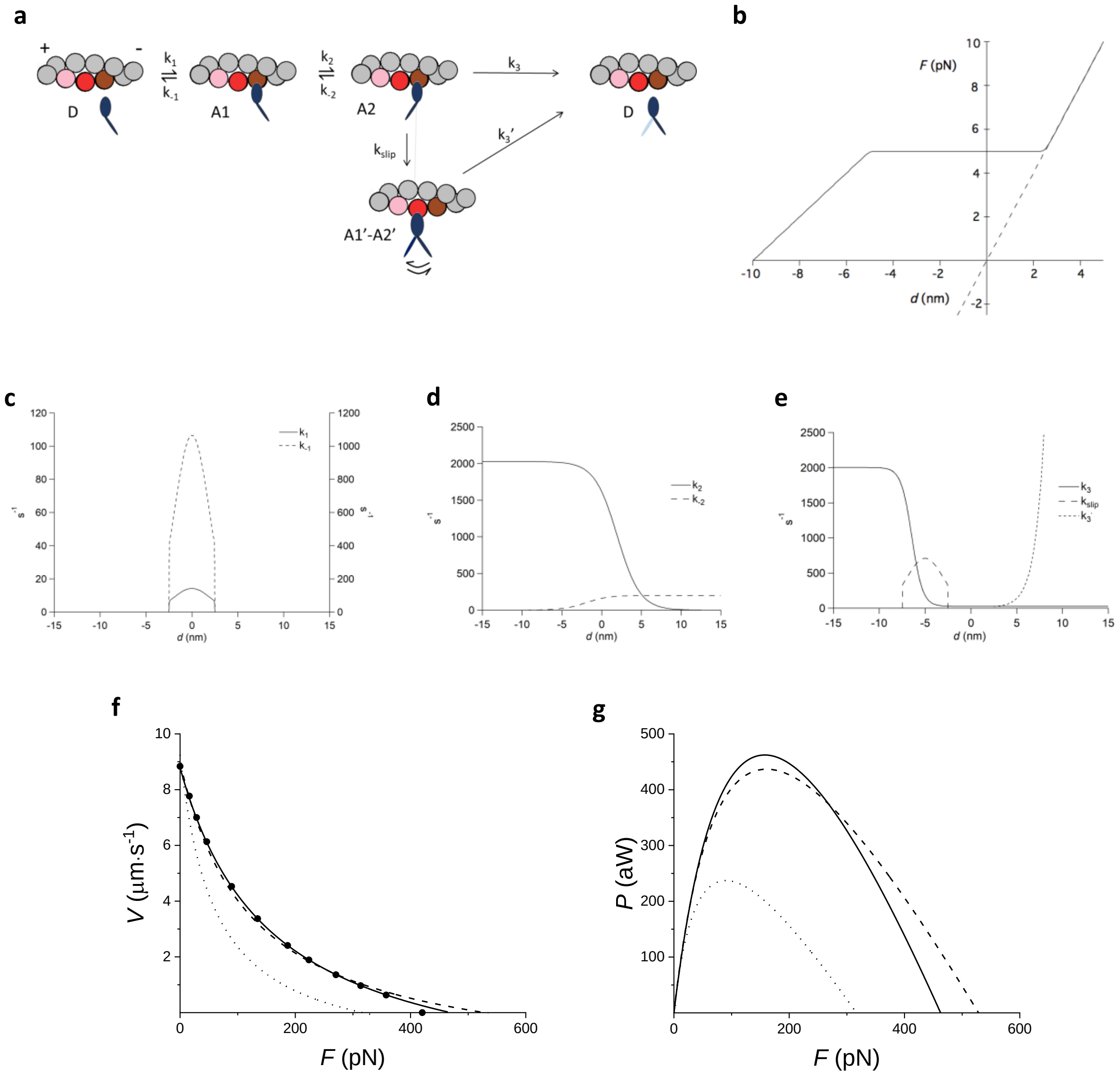

Supplementary Fig. 6. Kinetic model. a The kinetic scheme, with three states of the myosin motor (blue): D, detached; A1 and A2, attached to an actin monomer (brown). The scheme includes the possibility that during shortening the motor attached in the A2 state slips to the next actin monomer farther from the centre of the sarcomere (red) within the same ATPase cycle. The probability of a second slipping to the pink monomer is limited to 1/10 of that of the first slipping. Slipping is required to fit the maximum power and maintain the ATPase rate at P_{max} ~3 times that at F_0[2,3]. **b** Force profile of the A1 (dashed line) and A2 (continuous line) states as a function of *d*, the relative position between the motor and the actin monomer; at *d* = 0 the motor is in A1 state and generates zero force. **c-e** Dependence of the rate functions for the state transitions on *d*. Where the transition is reversible, k_i is the forward and k_{-i} is the backward rate constant. The corresponding equations are listed in Supplementary Table 1. **c** step 1, attachment reaction, k_1 continuous line, k_{-1} dashed line. **d** Step 2, force generating transition, k_2 continuous line, k_{-2} dashed line. **e** Step 3, detachment reaction, k_3 continuous line. The slipping of A2 to the next actin farther from the centre of the sarcomere is governed by k_{slip} which becomes relevant around -3 nm (dashed line in **e**). Following attachment to the second actin the fractional occupancy of states A1' and A2' riequilibrates according to step 2 transition kinetics. Detachment from an attached state following slipping (step 3') occurs with a rate constant k_3' (dotted line in **e**), which for A2' has the same strain dependency as k_3 (k_3' is masked by

k_3), while for A1' rises exponentially for $d > 2.5$ nm. One ATP is split whatever is the path leading to detachment (either step 3 or 3'). **f** Simulation of the force-velocity (*F-V*) relation of fast mammalian muscle. The experimental *F-V* relation (filled circles) is calculated for the half-sarcomere at full overlap (294 motors available) from the data of fast mammalian muscle at room temperature[4,5]. The continuous line is Hill's hyperbolic equation[6] fitted to the results of the model simulation with a series compliance similar to that in situ (0.01 $nm \cdot pN^{-1}$). The dashed line is Hill's fit of the results of the simulation with all the parameters as defined in the previous simulation and a series compliance as that of the trap (3.7 $nm \cdot pN^{-1}$). The dotted line is the Hill's fit of the results of the simulation with all the parameters as the dashed line and the force corrected for the random orientation of the motors. **g** *P-F* relations calculated from Hill's fits in **f** identified by the same line code.

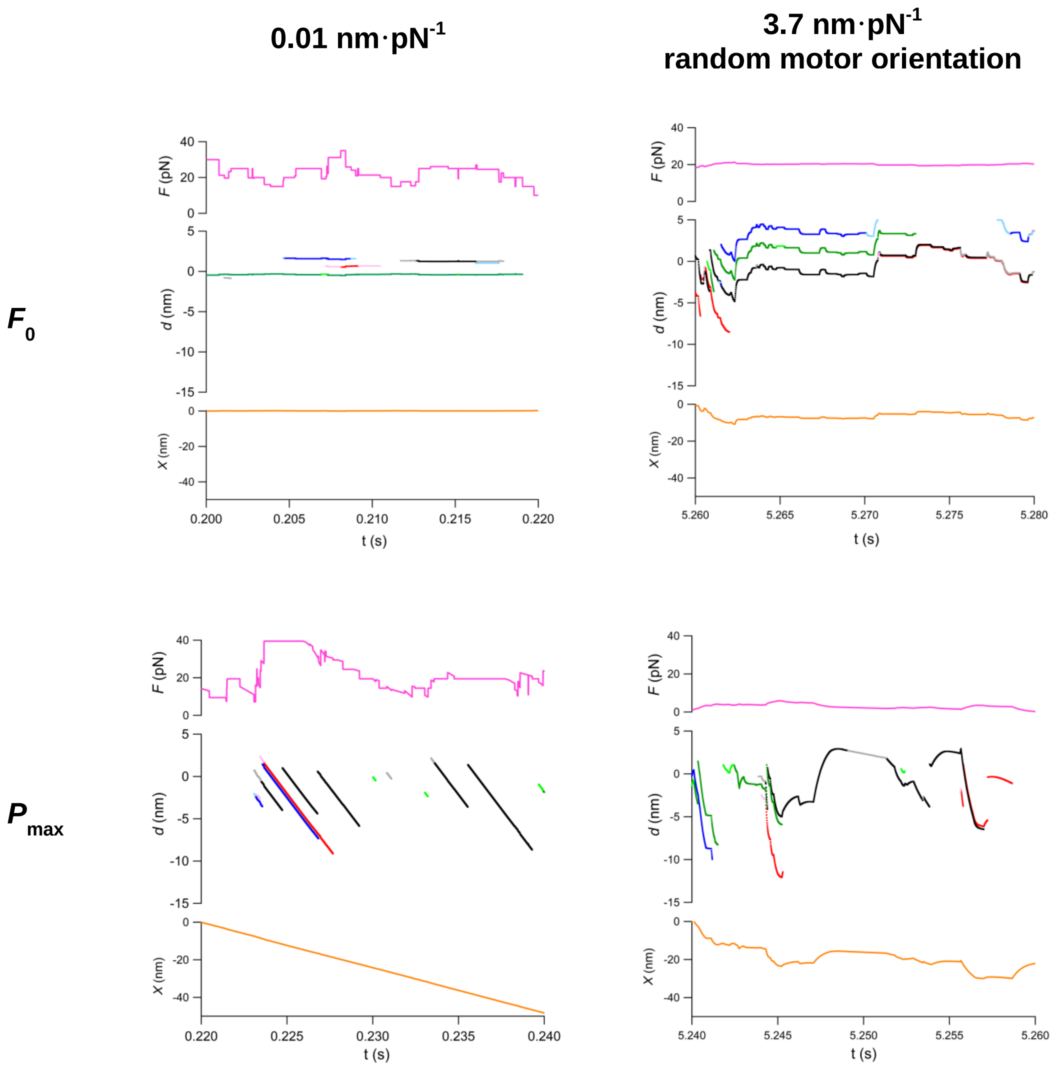

Supplementary Fig. 7. 20 ms time windows of the simulated traces of force (*F*) and axial position (*d*) of four motors from an ensemble of 16. Mode: position clamp. Upper row, at the plateau of isometric force (F_0); lower row, during sliding at the velocity for maximum power (P_{max}); left column, series compliance 0.01 nm·pN^{-1}; right column, series compliance 3.7 nm·pN^{-1} and random motor orientation. The four motors are color coded and the state is identified by the color tone: A2 state, red, dark green, black and blue; A1 state, pink, light green, gray and cyan respectively.

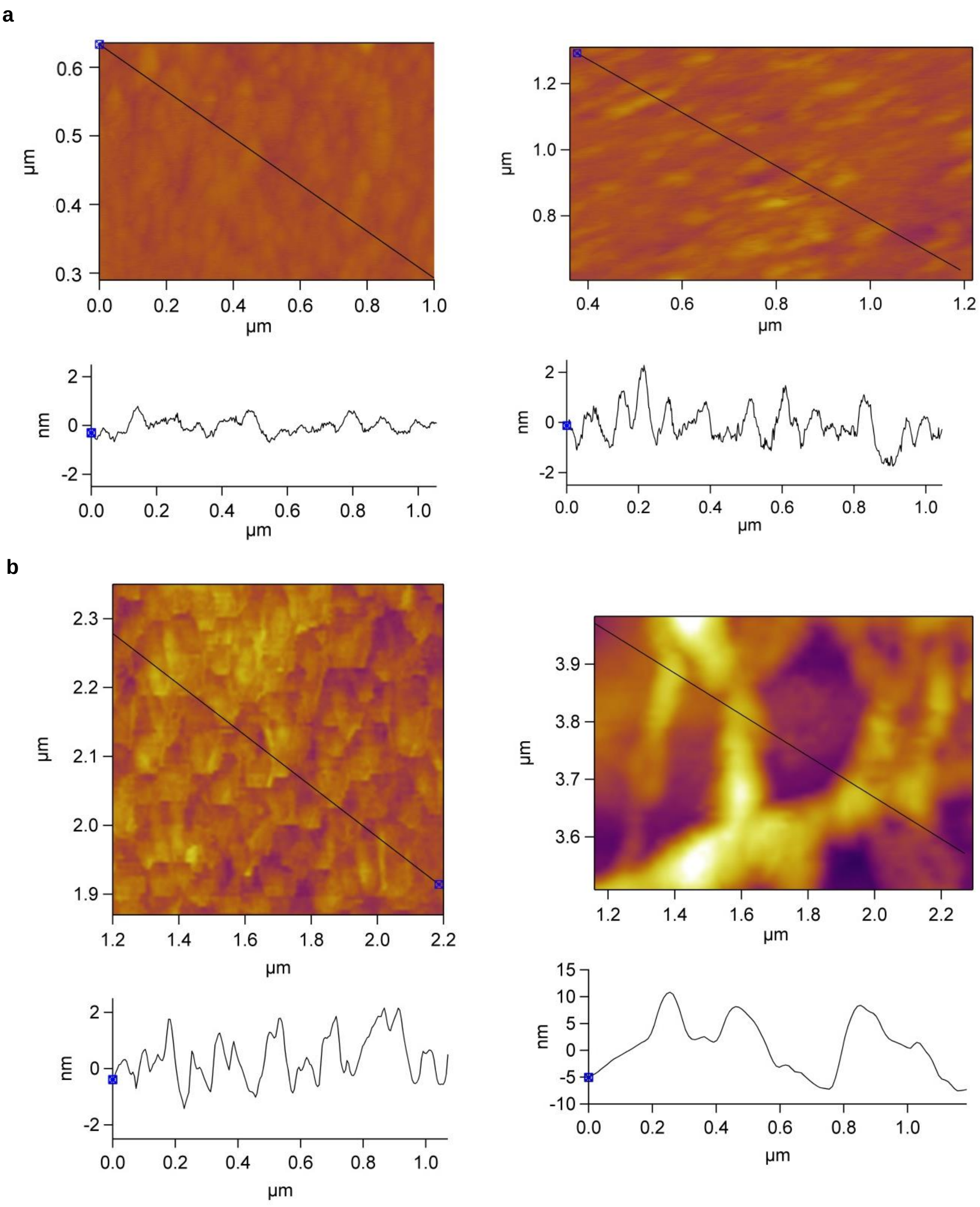

Supplementary Fig. 8. AFM image of the HMM support. AFM images in dry conditions, before (left column) and after (right column) the deposition of HMM (30 $\mu g \cdot ml^{-1}$) on the surface of a single mode optical fibre etched to a diameter of 4 µm and functionalised with a film of nitrocellulose 1% (w/v). **a** Scans of the lateral surface of the fibre mounted horizontally on the AFM support. The surface is perturbed by relatively narrow peaks of height <1 nm before HMM deposition (left) and ~2 nm after HMM deposition (right), which is consistent with a regular deposition of dried HMM. **b** Scans of the tip of the fibre mounted vertically on the AFM support. The surface is perturbed by wider peaks of height 3-4 nm before HMM deposition (left) and ~15 nm after HMM deposition (right), which indicates the formation of HMM aggregates. The blue squares mark the starting points in the image and in the corresponding profile.

k_1	$= (70 /\chi/\surd(2*\pi))*\exp(-d^2 /2*\chi^2)$<br>$= 0$	-2.5 nm $\le d \le 2.5$ nm<br>$\lvert d \rvert > 2.5$ nm
k_{-1}	$= (4816/\chi/\surd(2*\pi))*\exp(-d^2 /2*\chi^2)$<br>$= 0$	-2.5 nm $\le d \le 2.5$ nm<br>$\lvert d \rvert > 2.5$ nm
k_2	$= 2027*\exp(-0.7*(d-1.964))/(1+\exp(-0.7*(d-1.964)))$	
k_{-2}	$= 200*\exp(0.7*(d+2))/(1+\exp(0.7*(d+2)))$	
k_3	$= (1964*\exp(-1.7*(d+6.45))/(1+\exp(-1.7*(d+6.45))))+40$	
$k_{3'}$	$= (1964*\exp(-1.7*(d+6.45))/(1+\exp(-1.7*(d+6.45))))+40$<br>$= 25+(\exp(d-2.1)*5+\exp(d-2.1)*5)$	$d \le 2.5$ nm<br>$d > 2.5$ nm
k_{slip}	$= (3500 /\chi/\surd(2*\pi))*\exp(-(d+5)^2/2*\chi^2)$<br>$= 0$	-7.5 nm $\le d \le -2.5$ nm<br>$d < -7.5$ nm, $d > -2.5$ nm

Supplementary Table 1. Equations expressing the *d*-dependence of the rate constants of the forward and backward transitions used in the model. $\chi = 1.8$ nm.

Supplementary References

1. Nishizaka, T., Seo, R., Tadakuma, H., Kinosita, K. Jr & Ishiwata, S. Characterization of single actomyosin rigor bonds: load dependence of lifetime and mechanical properties. *Biophys. J.* **79,** 962-74 (2000).

2. Piazzesi, G. & Lombardi, V. A cross-bridge model that is able to explain mechanical and energetic properties of shortening muscle. *Biophys. J.* **68,** 1966-79 (1995).

3. Caremani, M., Melli, L., Dolfi, M., Lombardi, V. & Linari, M. The working stroke of the myosin II motor in muscle is not tightly coupled to release of orthophosphate from its active site. *J. Physiol.* **591,** 5187-205 (2013).

4. Ranatunga, K.W. Temperature-dependence of shortening velocity and rate of isometric tension development in rat skeletal muscle. *J. Physiol*. **329,** 465-83 (1982).

5. Ranatunga, K.W. The force-velocity relation of rat fast- and slow-twitch muscles examined at different temperatures. *J. Physiol.* **351,** 517-29 (1984).

6. Hill, A.V. The heat of shortening and the dynamic constants of muscle. *Proc. Roy. Soc. B.* **126,** 136-195 (1938).
