# Supplementary material for: A myosin II nanomachine mimicking the striated muscle
Source: Nat Commun. 2018 Aug 30;9:3532. doi: 10.1038/s41467-018-06073-9 (PMC6117265; doi:10.1038/s41467-018-06073-9)
Supplement: Supplementary file 3 — Description of Additional Supplementary Files [file 41467_2018_6073_MOESM3_ESM.docx]

**Description of Additional Supplementary Files**

File Name: Supplementary Movie 1

Description: IVMA test on the lateral surface of the etched optical fibre. Related to the experimental procedures. Sliding of TRITC-phalloidin labelled actin filaments on the HMMcovered lateral surface of an optical fibre etched to a diameter of ~4 μm and functionalised with nitrocellulose. [ATP] = 2 mM, temperature 23 °C.
