# Supplementary figures and images for: A myosin II nanomachine mimicking the striated muscle
Source: Nat Commun. 2018 Aug 30;9:3532. doi: 10.1038/s41467-018-06073-9 (PMC6117265; doi:10.1038/s41467-018-06073-9)

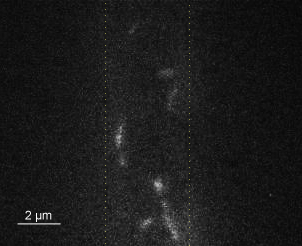

Supplement: Supplementary file 4 — Supplementary Movie 1 [file 41467_2018_6073_MOESM4_ESM.gif]
